# A Real-Time GNSS-R System for Monitoring Sea Surface Wind Speed and Significant Wave Height

**DOI:** 10.3390/s22103795

**Published:** 2022-05-17

**Authors:** Jin Xing, Baoguo Yu, Dongkai Yang, Jie Li, Zhejia Shi, Guodong Zhang, Feng Wang

**Affiliations:** 1 School of Electronic and Information Engineering, Beihang University, Beijing 100191, China; jinxing@buaa.edu.cn (J.X.); edkyang@buaa.edu.cn (D.Y.); Lzzz18@buaa.edu.cn (J.L.); zy2002418@buaa.edu.cn (Z.S.); zhang2017@buaa.edu.cn (G.Z.); 2 State Key Laboratory of Satellite Navigation System and Equipment Technology, Shijiazhuang 050200, China; yubg@sina.cn

**Keywords:** GNSS reflected signals, monitoring system, sea conditons

## Abstract

This paper presents a monitoring system based on Global Navigation Satellite System (GNSS) reflected signals to provide real-time observations of sea conditions. Instead of a computer, the system uses a custom-built hardware platform that incorporates Radio Frequency (RF), Field Programmable Gate Array (FPGA), Digital Signal Processing (DSP), and Raspberry Pi for real-time signal processing. The suggested structure completes the navigation signal’s positioning as well as the reflected signal’s feature extraction. Field tests are conducted to confirm the effectiveness of the system and the retrieval algorithm described in this research. The entire system collects and analyzes signals at a coastal site in the field experiment, producing sea surface wind speed and significant wave height (SWH) that are compared to local weather station data, demonstrating the system’s practicality. The system can allow the centralized monitoring of many sites, as well as field experiments and real-time early warning at sea.

## 1. Introduction

Environmental data collecting that is efficient and accurate is critical for operations including maritime production, resource preservation, disaster monitoring, and marine warfare. Traditional sea condition monitoring methods include buoys [1], current meters [2], and survey vessels [3,4]. However, the detection range is limited, and only point or line sea surface information can be collected, and the operating method is severely limited by maritime weather circumstances. Microwave remote sensors such as scatterometers [5], altimeters [6], and synthetic aperture radars [7] use a single-base technique for active detection, which allows for a wide measurement range but comes at a high cost, limiting their utility. As an emerging remote sensing technology, Global Navigation Satellite System-Reflectometry (GNSS-R), which was originally introduced by Martin-Neira [8] for ocean mesoscale altimetry, has been greatly extended to a wide range of applications. GNSS-R signals are used to retrieve the parameters of the Earth from the reflection surface, such as sea surface height [9,10,11,12], wind speed [13], sea ice [14,15,16], significant wave height [17], vegetation [18], soil moisture [19], target detection [20], and snow [21]. Classified from the receiver placement position, it can be divided into spaceborne [22], airborne [23], shipborne [24], shore-based [25]. Because GNSS-R technology makes full use of existing navigation satellite signal resources, and does not require the construction of transmitting equipment, the measurement device’s size, power consumption, and cost are considerably decreased. With the ever-improving GNSS system, a vast number of on-orbit navigation satellites ensures a huge number of accessible signal sources, making large-scale, low-cost, high-spatial-resolution surface detection possible. At the same time, the L-band employed by the GNSS system is less attenuated in the cloud and rain layer than the Ku, X, C, and other bands used in traditional remote sensing, making wind and wave remote sensing possible even in extreme weather such as typhoons [26]. The GNSS-R technology corrects the flaws in traditional remote sensing technologies and has significant research value. Because the reflected signals differ from the signal directly received from the satellite in terms of delay, Doppler shift, power strength, and polarization, they can be used to characterize the Earth’s surface [27,28,29,30,31,32]. These differences are dependent on the scattering surface’s geophysical qualities, and so may contain information about the surface geophysics. This paper investigates the conditions of the oceans and proposes a real-time system to retrieve sea parameters.

At present, the process of sea conditions retrieval using GNSS-R is cumbersome. Usually, the collected reflected signals are processed by correlation operation, and then the data is transmitted to the host computer for retrieval computation of physical quantities. The whole process often requires the coordination of multiple platforms, which consumes a lot of manpower and material resources [33,34,35,36]. The environmental characteristics of the remote sensing field also have a higher demand for lighter and more convenient equipment. Many GNSS-R sensors have been designed, and used to estimate geophysical features [37,38]. Custom GNSS receivers based on application-specific integrated circuits (ASIC) or FPGA make up the hardware of classic GNSS-based passive radars [39]. They typically necessitate a personal computer (PC) with enough capacity to retain a substantial quantity of data, which is not portable for outdoor experimenters. Furthermore, their large and heavy setup prohibits them from being used in the field. To solve these issues, several researchers have managed to develop equipment that is small and portable. The design and development of a GNSS passive radar for the classification of lands is presented in [40], which is intended for small UAVs. A prototype of an FPGA-based real-time GPS reflectometer was described in [41,42]. GEC-computer Plessey’s chip, which includes 12 correlators, is utilized in NASA’s Langley Research Center’s test equipment. The gadget provides 1 ms coherent integration results with a sampling rate of 5.7 MHz and a temporal resolution of 500 ns [43]. The German GFZ gadget is based on OpenGPS receivers’ software and hardware development, as well as GEC-Plessey processors, which can analyze four satellites’ reflected signals at the same time with a 500 ns delay interval [44]. The GOLD-RTR (GPSopen-loopdifferentialreal-timereceiver) receiver from IEEC processes digital signals using FPGAs, has 10 parallel computing channels and three soft-core processors with a 50 ns delay interval, and may be dynamically adjusted for different satellite data channels [45].

The system developed in this paper is intended for real-time signal processing. Different from the mentioned receivers, the hardware board is utilized as a substitute for the host computer in this paper to achieve the collecting and processing of reflected signals with a structure of Raspberry Pi, and it takes full use of its advantages of compact size, low power consumption, and cheap cost. It is a real-time retrieval prototype with an integrated design. This study offers a real-time performance of the sea surface wind speed and wave height system, including Beidou and GPS system, in addition to this benefit, the system with the transmission module, may remotely observe the scene sea conditions after collecting a signal on the scene. The study of the availability of the sea conditions monitoring system and the algorithm in conjunction with hardware resources has significant practical implications and application prospects in the field of remote sensing on the sea, and can serve as a powerful supplement to traditional remote sensing methods.

Section 2 highlights the primary design criteria that influenced the initial phase of development, including the sensor’s lightweight and the system’s real-time need. The hardware architecture is described in Section 3 along with the arrangement of the components that were incorporated into the case. The data transmission between FPGA and DSP, which is crucial in signal processing, is the most significant aspect. It also goes through some of the specifics of the data storage local server group. The background of such a discipline is briefly discussed in Section 4 to provide further proof of the general process of the algorithm for retrieving sea conditions. As a result, Section 5 begins with a description of the field results of sea state monitoring at a sea location. Finally, Section 6 brings the article to a close by discussing several unresolved concerns, as well as future advances and applications of this research.

## 2. Requirements

The purpose of this research is to develop and test a coastal sensor capable of measuring GNSS reflected signals. Sea condition parameters such as sea surface wind speed and significant wave height can be retrieved using these signals. The system undergoes field tests. The structural and control components must be complete, and the system must be able to test the application at high wind speeds. The total power consumption should be less than 20 W, and it incorporates a reverse power supply safeguard mechanism. The system should have multi-channel correlation functions to output direct/reflected signal-associated value data, as well as support GPS and Beidou systems. It should also be able to achieve acquisition, tracking, and analyze GNSS direct signals and generate navigation and positioning data. The hardware component should be able to interpret navigation positioning results and signal correlation value data, extract observational measurements relevant to sea surface wind and waves, and then conduct sea surface wind speed and significant wave height detection using these observations. Finally, the system should be able to support 4G communication network transmission as well as wireless LAN transmission, and it should be able to determine the best method for uploading sea surface wind and wave detection data based on the network environment. In terms of data utilization, the system should offer data export and have real-time information storage functions.

## 3. Prototype Design

**Figure 1:**
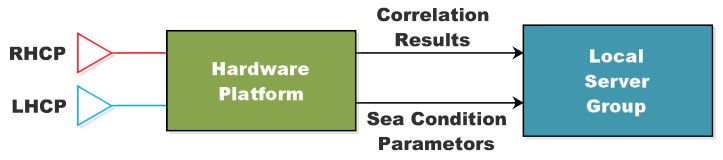
Information flow and the two layers of the prototype design.

The sensor’s design flow has been divided into two levels, as shown in Figure 1:

- the hardware platform;
- the local server group.

The hardware architecture, which includes the RF front-end, baseband processing module, and antennas, is defined initially. Following that, suitable GNSS signal processing algorithms must be designed in order to identify and evaluate the relative latency and amplitude of reflected GNSS signals. To achieve real-time performance of the entire system, the system opts for on-the-fly signal processing and parameter retrieval, then transmitting the results to the local server group, which has real-time requirements for the on-site hardware. High computational abilities are necessary for signal processing and retrieval processing. Finally, the correlation results, as well as the retrived sea conditions parameters, are uploaded to a server that users may access via the interface.

The essential elements of the sensor’s hardware platform were developed from the high-level application requirements described above. They are arranged by hardware components, Local Server Group implements, and functional validation in the subsections below.

### 3.1. Hardware Components

The essential hardware components of the on-board sensor are:

- the GNSS antennas;
- the commercial off-the-shelf (COTS) RF front-ends (FEs);
- the Field Program mable Gate Array (FPGA) stage;
- the digital signal processing (DSP) stage;
- the Raspberry Pi;
- the COTS communication module.

**Figure 2:**
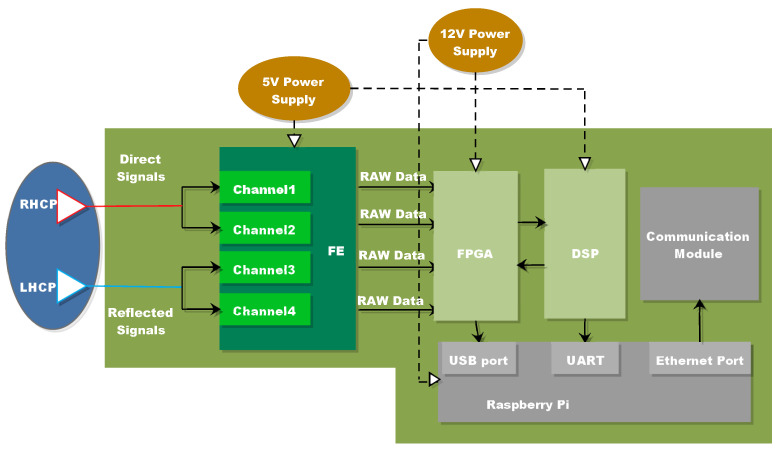
Hardware scheme of the sensor.

Figure 2 depicts the sensor’s functional block diagram, with the key hardware components and their connections indicated. While a standard low-cost hemispherical GNSS Right-Handed Circular Polarization (RHCP) patch antenna aimed at the zenith is sufficient to receive direct GNSS signals, reflected signals require a Left-Handed Circular Polarization (LHCP) antenna aimed at the nadir (the parameters are shown in Table 1). Two single-polarization antennas, compatible with GPS L1 and Beidou B1 frequencies, were developed to receive GNSS direct and reflected signals. 1575.42 MHz and 1561.098 MHz are the center frequencies, and the direct antenna gain is roughly 5 dB. Because the GNSS signal’s polarity will change after reflection, and the signal-to-noise ratio will drop dramatically, the characteristics of the chosen antenna are as follows, which may match the criteria of poor signal reception.

After the antennas, the RF front-end is the second step of the hardware platform. The MAX2769 RF chip, created by Maxim, is selected in the system after a thorough review of system performance, power consumption, cost, and other factors. With a total noise figure of less than 1.4 dB, the device employs an innovative low-power SiGe BiCMOS technology and includes a complete receiving connection within. Signal conditioning, RF down-conversion, filtering, and analog-to-digital conversion are all part of it. In the FE, the number of RF chains must be equal to the number of different signals. The four processing chains of the RF are used here. For the direct signal, two RF chains are used and coupled to the zenith-pointing antenna. The other two RF chains, on the other hand, are dedicated to the two LHC-polarized reflected signals and are connected to the nadir-pointing antenna’s two ports. The sampling frequency (16.369 MHz) produces a 2-bit quantized signal for Beidou and GPS system. A series of pin connections connects the RF front end to the backplane of the baseband processor module. On all four chains, the same clock reference must be used. Furthermore, the FE comes with a single 24 MHz TCXO reference. The onboard hardware is linked in Figure 2 as follows: the direct signal is supplied to Channels 1 and 3 in the FE as a reference, while the LHCP antennas are attached to the other channels. Furthermore, the raw data is transferred to the FPGA and DSP stages (described below) for further signal processing.

After the FE, a baseband processing module including FPGA and DSP stage is required to process the digital data. The signal is sampled by the high-speed A/D converter and then fed to the digital quantization module in the FPGA after being filtered and down-converted into intermediate frequency analog signals. The quantized information from the channels is transferred to the direct and reflection channels in FPGA for real-time data processing. To perform satellite acquisition and tracking, navigation positioning, satellite status updating, and other tasks, the direct channel collaborates with DSP. The satellite reflected signal’s reflection channel is set up to actualize the delay and then acquire varied time delays and correlation results.

The XC7325T chip from Xilinx’s Kintex-7 family is used, which allows for simultaneous operation of direct and reflected signal multi-channels. It uses less power and less cost, making it easier for the system to do multi-point networking. The TMS320C6747 processor from Texas Instruments is used for DSP, and it has a high computing speed and outstanding peripheral interfaces, and it fulfills the baseband signal processing capability in conjunction with FPGA.

**Figure 3:**
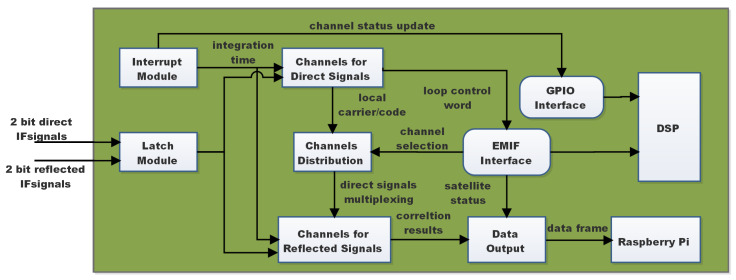
Principle block diagram of FPGA.

FPGA creates carriers and codes of various frequencies using digitally controlled oscillators and achieves the correlation operation of direct and reflected signals under the operation of DSP. DSP completes direct signal acquisition and loop judgment, controls FPGA synthesis of local carrier and PRN code, extracts positioning messages, and configures reflection channels. A total of 30 direct channels and 12 reflection channels have been created, allowing the satellite to be acquired and tracked while simultaneously processing the reflected signals. The direct and reflection channels are dynamically assigned, and the direct correlation result is given to DSP to help complete the tracking loop. The result of the reflection correlation is combined with satellite status information and sent to the Raspberry Pi via USB. Vivado Environment is used to write, test, and upload the application that runs on the FPGA. Furthermore, C++ with Code Composer Studio (CCS) completes the DSP application (Version 5.3). Direct signal processing and reflected signal processing are two of the key tasks of the multi-channel dedicated correlator based on FPGA. Figure 3 depicts the basic block diagram of the system.

The latch module buffers and re-encodes the digital intermediate frequency (IF) signal. For future correlation procedures, the re-encoded direct and reflected signals are fed into the direct and reflection channels, respectively. The interrupt module generates a 1ms integral clearing signal with the help of a counter, which adds up the correlation findings in the direct and reflections channels. DSP receives a status update signal through the General-Purpose Input/Output (GPIO) interface, which triggers the DSP interrupt program and performs the channel status update operation. The direct channel generates two local carriers with a 90° phase difference using a digital frequency synthesizer. I (in-direction) and Q (quadrature) signals are obtained by combining them with the direct intermediate frequency signal. The shift register and delay device create three local PRN codes of E (early), P (immediate), and L (late) with a 0.5 chip interval, and the 1ms correlation accumulation operation is performed using the direct I and Q signals, respectively. Additionally, the External Memory Interface (EMIF) can be used to send the operation result to DSP. To realize the acquisition and tracking of the direct signal, DSP’s carrier loop and code loop return the control word based on the result and alter the carrier and pseudocode generated by the direct channel to make it consistent with the received signal.

**Figure 4:**
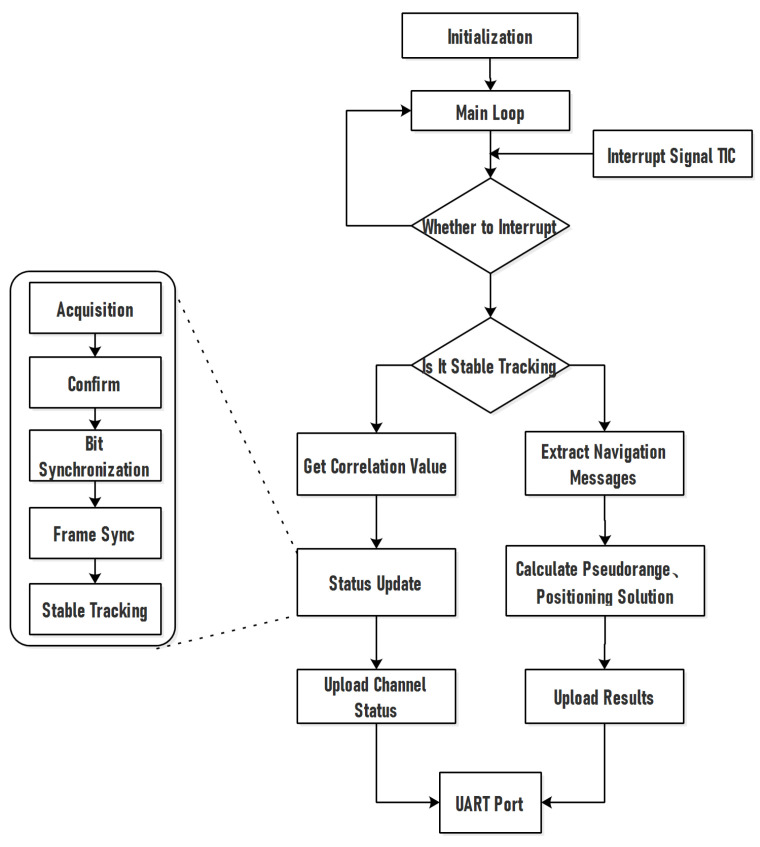
DSP signal processing flow.

As shown in Figure 4, DSP is the other step of the baseband processing module and is responsible for real-time processing of digital signals. It is ideal for creating complicated logic algorithms and system control due to its command system. DSP may build a closed signal tracking loop with FPGA in the baseband processing module, allowing it to acquire and track the direct signal while also controlling the distribution of the reflection channel. For direct signal processing, DSP uses the interrupt response method. With a 1s interval, the FPGA emits the Task Interrupt Control (TIC). When the TIC comes, DSP exits the main program’s waiting loop and executes the response program. The interrupt response software conducts two branches of status update and positioning solution, depending on the difference in the present condition of the channel.

- Status updating

When the channel’s tracking is not complete, the software switches to channel status updating. The system has a five-channel status of capture, confirmation, bit synchronization, frame synchronization, and stable tracking, and it employs FGPA’s correlation value information to determine the current loop’s signal lock. To ensure steady signal tracking, the channel status is jumped and updated according to the judgment result, the satellite number, signal-to-noise ratio, correlation waveform, and other information are communicated, and the carrier’s control word and code are returned to FPGA.

- Positioning

When the number of monitored satellites is higher than or equal to 4, this branch completes the extraction of the direct signal navigation message, calculates the pseudo-range of the satellite, and conducts the positioning solution of the navigation signal. If the current channel’s reflection signal computation has not been completed, an idle reflection channel is assigned to it, and the channel distribution code is created and delivered to FPGA.

**Figure 5:**
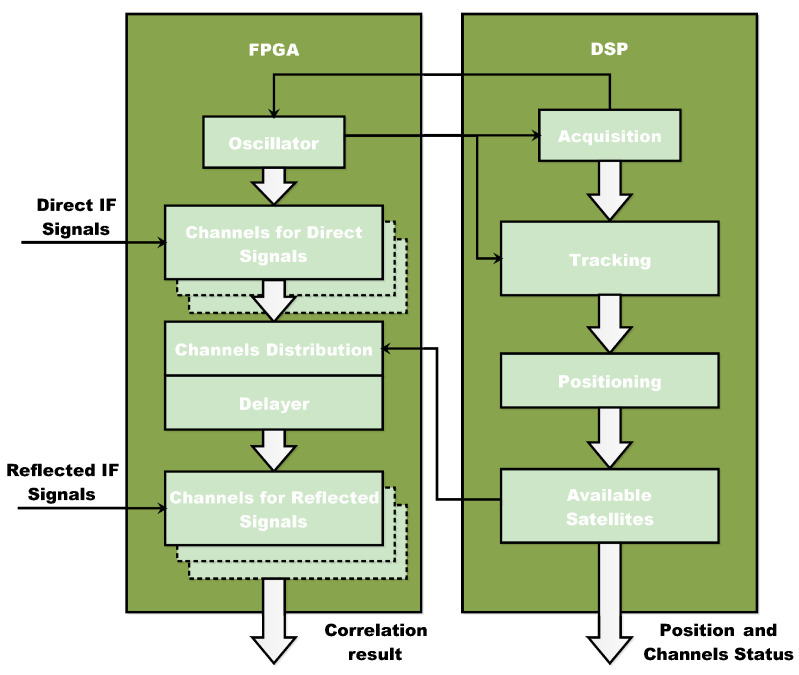
Connection between FPGA and DSP.

The data stream that is exchanged between DSP and FPGA in this article uses an EMIF. The FPGA can be used as the DSP’s external memory, allowing for high-speed data transfer through addressing. Figure 5 depicts the connecting procedure. The system uses a 12-bit address bus and a 16-bit data bus, with fixed addresses assigned to the data to be transferred. DSP delivers the target data address to the FPGA through the address bus, and FPGA connects the relevant data to the data bus via a state machine. After that, the data bus may be read and written by DSP and FPGA, respectively. The DSP interrupt signal is received through the GPIO interface. EMIF and GPIO wire is directly linked to FPGA backplane via pins.

**Figure 6:**
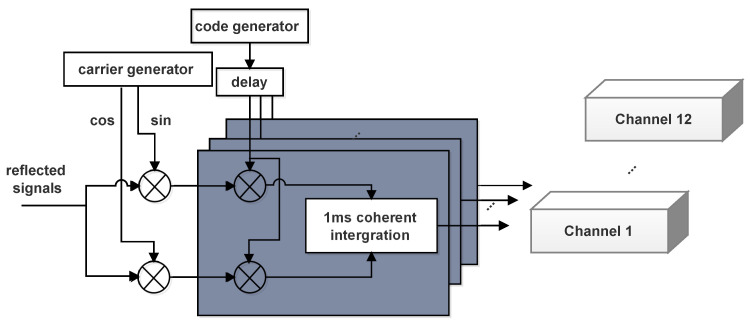
Channel structure of the reflection channels.

The reflection channel’s job is to carrier strip the reflected intermediate frequency signal, construct a time-delayed PRN code, and calculate the correlation value of the reflected signal with various time delays. Unlike the direct channel, the reflection channel lacks a local carrier and code generating module. To achieve signal multiplexing, it introduces the carrier and code created in the appropriate direct channel through dynamic distribution. The Doppler frequency shift between the reflected and direct signals may be ignored because the system is designed for shore-based applications. Through the frequency multiplier, the reflection channel acquires the chip delay control clock and 20 channels of time delay PRN codes at an interval of 0.25 chips and a range of −2 to 3 chips. For retrieval, the delay range and resolution are suitable. Each branch follows the same correlation procedure as the direct channel, resulting in a total of 20 delay correlation values and a correlation duration of 1ms. The structure of the reflection channels is shown in Figure 6.

**Figure 7:**
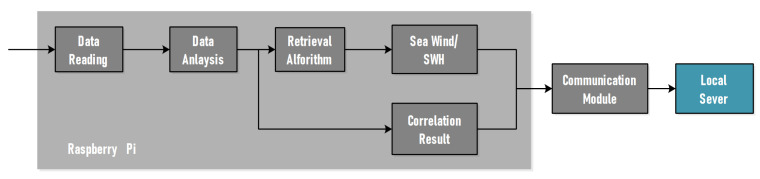
Retrieval unit scheme.

The retrieval unit, which contains a Raspberry Pi and a communication module, is the last component of the hardware platform. A desktop computer or an industrial computer is typically utilized for retrieval in the design of a typical system for sea condition monitoring. Although this method has a fast computation speed, it is expensive and consumes a lot of power in real applications, and it is restricted by size and power supply. To address the aforementioned flaws, the system employs Raspberry Pi as a stand-in for an industrial computer to perform the retrieval function of sea wind and waves. A Linux system may be installed on the Raspberry Pi, which has the advantages of compact size and low power consumption, making it ideal for long-term field observation. The retrieval unit is a Raspberry Pi 3B with a 1.2 GHz processor, four high-speed USB ports, Ethernet connections, HDMI connectors, and other features. The Raspberry Pi’s internal storage space is a 64 GB memory card, and the system’s overall performance matches the criteria. The process is depicted in the diagram below (Figure 7).

The data frame generated by the baseband processing module is read by the retrieval unit, which is linked to FPGA through a USB cable. Data communication necessitates the use of the system’s bottom layer interface and necessitates a high operating speed. In this research, C programming language is used to create USB drivers. It completes the USB communication between this portion and the device using the Linux system’s libusb package, which does data reading. After obtaining the correlation data, the data must be further examined to acquire the correlation results and status information for each reflection channel.

The data analysis program’s job is to convert binary data to decimal, analyze the data frame according to the communication protocol and extract the reflected signal’s feature using the retrieval method. The data analysis application is performed in Python 3 since Raspberry Pi has a high level of support for Python language and additional mathematical operation libraries. The software looks for the effective reflection channel in the data frame header first. If the channel is closed, the further processing is skipped. Information such as PRN, elevation angle, and I/Q value of signals may be derived by evaluating the content of the data frame.

To conduct wind and wave height retrieval results, we employ a technique based on Interferometric Complex Field (ICF) autocorrelation time, which is discussed in the following section. The correlation sequence is fitted with a Gaussian curve to determine the correlation time necessary for retrieval. The library is used by the application to finish the fitting function operation. To save resources, the channel might be closed. Finally, the results and raw data are transferred to a local server group for data visualization and storage.

As discussed above, the system components were integrated within a suitable case once the functional architecture was defined. In contrast to typical equipment, the field must guarantee that the equipment is lightweight, compact, and simple to install. The air humidity is rather high on outdoor and open-air occasions, especially at the coastal site, and it is vital to maintain the airtightness of the hardware platform. The casing was created with the intention of being readily opened for quick inspections and maintenance.

The sensor prototype measurements are 360 mm, 260 mm, 180 mm (length, width, and height) with a weight of less than 4 kg after being designed and constructed in this manner. The hardware platform is attached to the bottom board in order to be light and small.

**Figure 8:**
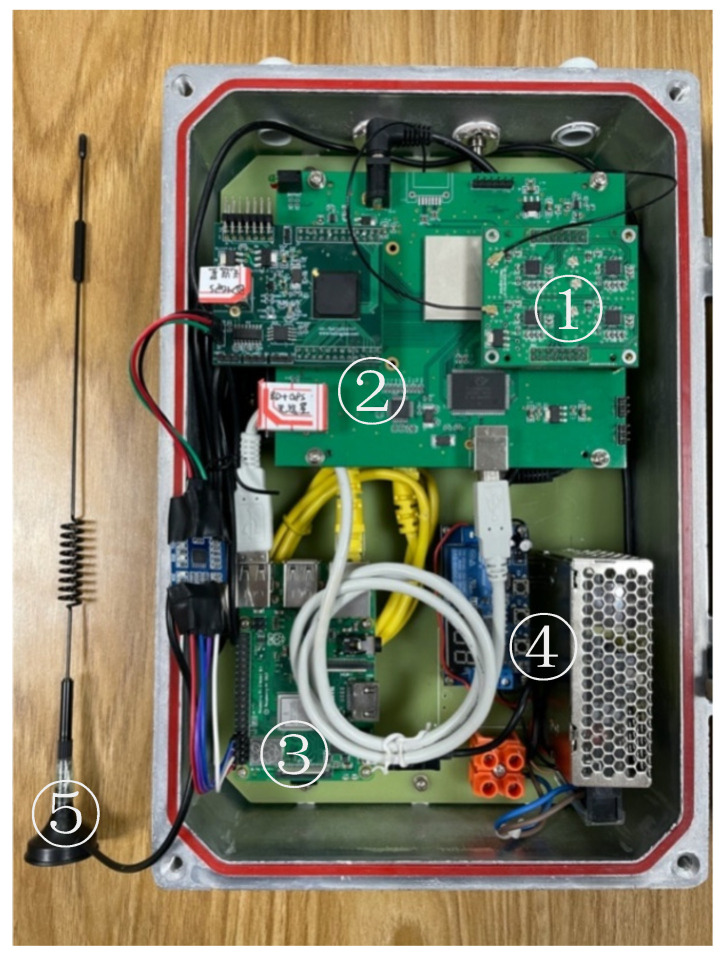
Physical map in the packaged case.

Each component can be placed in a stainless steel container (as shown in the Figure 8). Outside, LHCP and RHCP antennas are built with a cable connection via the case’s hole. The following are the case’s individual components:

(1) A four-channel radio frequency front-end module that can gather direct and reflected signals from Beidou and GPS systems.
(2) The correlation power and positioning result are output by the baseband processing module, which is made up of FPGA and DSP.
(3) Raspberry Pi, to finish data processing and uploading activities.
(4) The power supply module, including a voltage transformer, which supplies the whole system with electricity.
(5) A communication module with an antenna that allows for LTE wireless data transmission.

### 3.2. Local Server Group Implements

**Figure 9:**
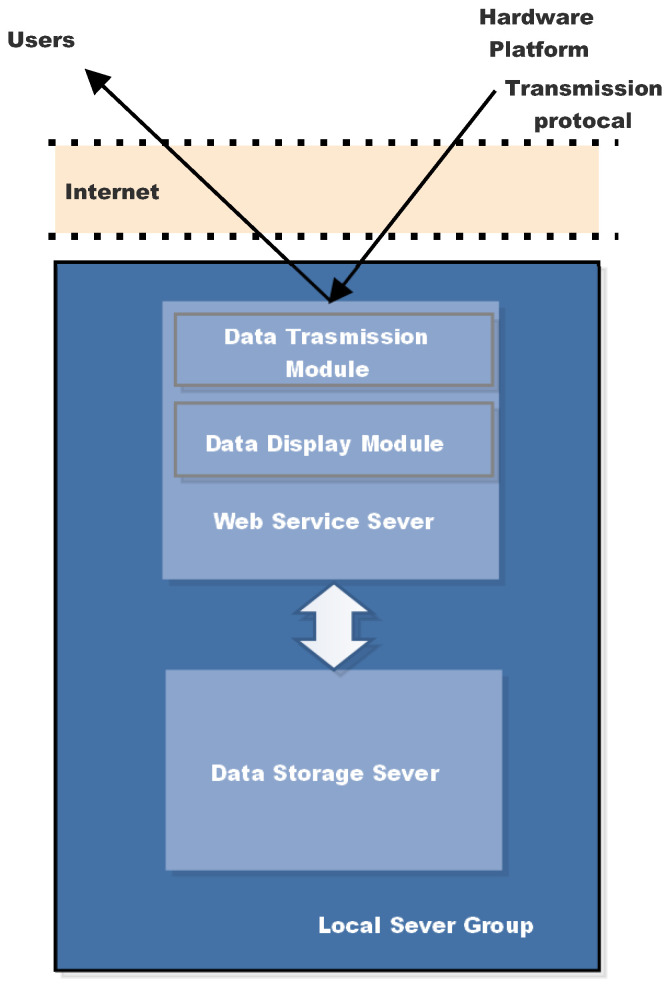
Local Server Group scheme.

As shown in Figure 9, it is implemented in this system employing servers that have been designed. Web Service Server and Data Storage Server are the two servers that make up the Local Server Group. By using the URL on the website, applications designed for users can obtain monitoring information. Web Server recognizes the data transferred from the field based on the transmission protocol: channel number, PRN number, elevation angle of the satellite, azimuth angle of the satellite, the number of the correlation value, delay window, direct I/Q value and reflect I/Q value. Moreover, sea state information is acquired by recognizing different fields. The Local Server Group is formed distant from the sea site to gather, standardize, store, and transfer data as the last portion of the system that is directly related with users. The design must be standardized, rational, and lightweight as much as feasible in order to meet the demand. The data acquired by the hardware is used to develop data transmission and storage standards.

The Web Service Server runs on Windows Server 2008, which includes network connections for both hardware and users. A number of Web Application Programming Interface (APIs) are built using the C# language for each piece of equipment to contact the server in order to realize data transfer and requests. On the sea site, bundled field equipment is placed, which connects to the Web Service Server through the Internet. Furthermore, using the Web API, real-time data is uploaded and saved in the Data Storage Server. The data reading, data analysis, and data processing functions are all possible with the calling interface. When the Web Service Server gets a request from a user, it connects to the Data Storage Server to retrieve the information. Finally, the results are presented on the internet, allowing for real-time monitoring of the sea status.

The Data Storage Server does not have any external network services, which enhances the data’s security to some level. It simply uses the local area network to interact with the Web Service Server. On the Centos 7.6 operating system, a MySQL 5.6 database was installed, and external hard drives were mounted to expand data storage capacity. Based on the data fields, data tables were formed, and a relationship model between the tables was established. The original correlation data and product data are kept in the design for further data inspection and research investigation.

The correlation data is 64 MB in size. The data is separated into numerous 1 MB data files to ensure the real-time efficiency of distant transmission. When the server gets the data files from the scene, it will combine the tiny files, ascertain the device number, and extract legitimate data using the satellite channel number as a guide. Analyze the data based on the satellite PRN number and compress it for storage in order to improve the usage rate.

An exhaustive test campaign was conducted in the laboratory to validate the prototype’s functionality. To test the hardware platform, we verified the sensor in a controlled environment, using all four channels to receive real RHCP signals from the antennas. In any case, such tests were required from a functional standpoint in order to verify the functionality of the channels in the hardware platform when linked to the antennas and to detect any distortions in the received signals.

We were able to validate all hardware components, as well as the software routines that implemented the usage modes and grabbing capability, first. In the test, The RCHP is installed on the building’s roof, which is open to the elements. A four-way power splitter divided the same GNSS signal and transmitted it to the four channels. Several test metrics were used in the analysis and validation process, including the digitalized signal’s power spectral density (PSD) and an estimate of the signal-to-noise ratio (SNR). The sensor passed all of the tests and confirmed its capacity to appropriately process the signal in all scenarios. The interaction of the data stream between the FPGA and the DSP is typical. In all of the tests, the receiver was able to correctly capture and track all produced satellite signals, according to the post-processing analysis.

**Figure 10:**
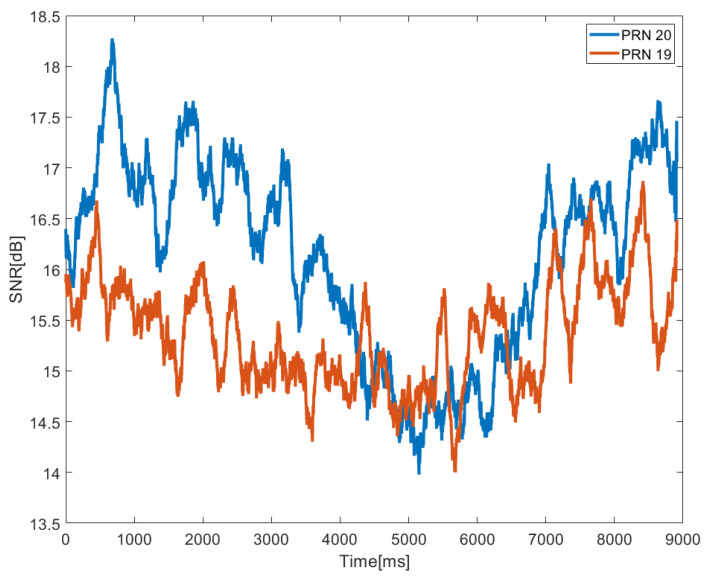
SNR evaluation for PRN20 and PRN 19, obtained during a test using real signal.

Figure 10 depicts the estimated SNR for two monitored satellites processing sample streams at the output of the four channels as an example. The SNR is calculated and is a reliable indicator of the received signal quality. It demonstrates that the values of SNR measured on samples taken from the hardware platform are presented in blue and red lines, respectively, indicating the available PRN 20 and 19 in the GPS system. Both signals are gathered and analyzed in 9 milliseconds in real-time reflection channels. The reflection channels are shown to have a constant collecting ability over the processing time.

**Figure 11:**
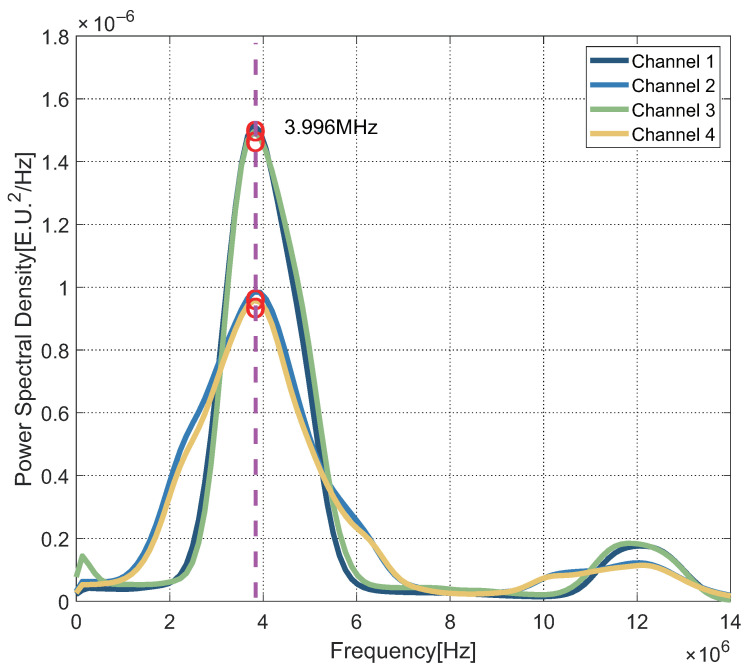
Power spectral densities of the signals at the four channels of FE.

In Figure 11, another example of the in-lab test results is reported. Here, the estimated PSDs of the IF signals are shown. In particular, both Beidou(B1I) and GPS(L1) system are shown. The processed signal comes from the FE. After spectrum analysis, as shown in the figure, it shows the intermediate frequency signal, 3.996 MHz, which is used for subsequent signal processing. In the 4 channels of the signal input, channels 1, 3 are used for the Beidou system, and the other two are GPS. The GPS signal is stronger and more evident in the bandwidth center. It can be explained as the Beidou signal has a wider bandwidth and the power are dispersed near the IF signal frequency point.

## 4. Sea Surface wind Speed and SWH Retrieval Algorithm from Reflection Measurement: A Background on the Discipline

The basic concepts of sea wind speed and significant wave height estimation using GNSS reflectometry are briefly reviewed in this section. To motivate the implementation decisions chosen in the prototype design, a study of this discipline’s theoretical basis is required.

When electromagnetic waves collide with the sea surface, they interact with the surface and disperse.

The reflected signals from calm seas exhibit significant coherence. A rough surface, on the other hand, scatters an incident signal in all directions, with intensity and phase varying depending on the scattering angle. Specular scattering reduces as the sea surface grows rougher, but diffuse scattering rises, resulting in the majority of reflected signals being incoherent. Correlation time [46] can be used to describe the correlation of GNSS reflected signals.

The correlation time and significant wave height sensitivity features of the GNSS reflected signal received by the shore-based platform were investigated and studied by Soulat [47]. They proposed using the ICF of the direct signal and the reflected signal to obtain significant wave height, assuming that the sea surface height follows a Gaussian distribution. The majority of the signals received originate from the Fresnel zone, which is located in the water near the specular point, due to the low intensity of the scattered signals.

**Figure 12:**
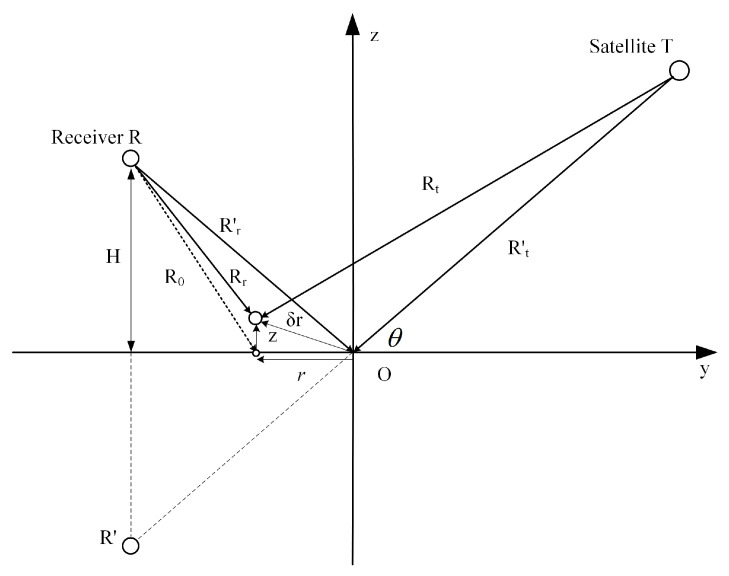
Bistatic geometry of the coastal GNSS-R scattering.

The complex correlation time series $F_{R}\left(t\right)$ and $F_{D}\left(t\right)$ correspond to the maximum correlation result of GNSS reflected and direct signals, respectively. The bistatic geometry of the coastal GNSS-R scattering is shown in Figure 12.

Where $R_{t}$ and $R_{r}$ are the vector from the navigation satellite and receiver to the scattering point. $R_{t}^{\prime }$ and $R_{r}^{\prime }$ are the vectors from the navigation satellite and the receiver to the specular reflection point. $R_{0}$ is the vector from the receiver to the projection point of the scattering point on the sea surface. $\delta_{r}$ is the vector from the specular reflection point to the scattering point. z and r are the vertical and horizontal displacements of the scattering point relative to the mirror point. H is the height of the equipment. Here, $r^{\prime }=R_{r}^{\prime }$, ${\hat{r}}_{⊥}^{\prime }$ is the horizontal unit component of $R_{r}^{\prime }$. Based on the geometric relationship shown above and the explanation of the parameters, the expression of the interference complex field is obtained as [48]:

(1) $$F_{I}\left(t\right)=F_{R}\left(t\right)/F_{D}\left(t\right)\approx ik\frac{e^{i2kH\sin\theta }}{r^{\prime }}\cdot \int MRe^{{i\left(-2kz\sin\theta -q_{⊥}\cdot r+\frac{k}{2r^{\prime }}\cdot r_{⊥}\cdot r\right)}^{2}}dS$$

In $M=\sqrt{g(r,z)}\chi \left(r,z\right)$, χ(r,z) is the ambiguity function. g(r,z) is the antenna gain, *k* is the wave number of the carrier. R is the sea surface reflection coefficient. θ is the elevation angle of the satellite.

Based on the Elfouhaily spectrum, the relationship between correlation time of the sea surface and the SWH can be obtained as:

(2) $$\tau_{z}=a_{s}+b_{s}*\mathrm{SWH}$$

where $a_{s}$, $b_{s}$ are undetermined coefficient, which depends on the sea condition, and the effective correlation time of ICF is defined as:

(3) $$\tau_{F}^{\prime }\equiv \tau_{F}\sin\theta =f\left(\mathrm{SWH}\right)$$

The expression of $\tau_{F}$ is:

(4) $$\tau_{F}^{\prime }\approx \frac{\lambda }{\pi }\left(\frac{a_{s}}{\mathrm{SWH}}+b_{s}\right)$$

In order to describe the SWH more accurately in the system, an offset parameter ${\mathrm{SWH}}_{0}$ and a scale parameter *p* are introduced as:

(5) $$\mathrm{SWH}\approx {\mathrm{SWH}}_{0}+p\frac{a_{s}}{\tau_{F}^{\prime }\pi /\lambda -b_{s}}$$

where *p*, $a_{s}$ and $b_{s}$ are undetermined coefficients, which are set according to the specific location and the installation height of the device. The sine of the elevation angle is processed separately to obtain a more accurate result, where a power function empirical model is established.

(6) $$\mathrm{SWH}\left(t\right)=a\tau_{F}^{b}\left(t\right)\sin^{c}\theta \left(t\right)+d$$

*a*, *b* and *c* are undetermined coefficient in the empirical model.

In terms of the sea wind, there is a strong relevance between sea surface wind speed and significant wave height. Wind speed is carried out by establishing an empirical model of the correlation time and wind speed. However, the response of sea waves to sea wind usually has a lag. A delay parameter δ is added to the SWH retrieval model to establish a wind speed retrieval model [49]:

(7) $$w_{s}\left(t\right)=a^{\prime }\tau_{F}^{b^{\prime }}\left(t+\delta \right)\sin^{\prime }\theta \left(t\right)+d^{\prime }$$

## 5. Results of an In-Field Test

The collected results and findings are summarized in this part, which focuses on the practical use of the developed sensing system on a coastal location. To evaluate the prototype’s performance, a 25-day data gathering effort was conducted. The goal of the test campaigns was to show that the prototype could deliver the sea wind speed and SWH using the retrieval technique described earlier in this section. A brief overview of the maritime sites chosen as case studies is provided. The accuracy of the sea wind and SWH obtained in the system is then demonstrated, and the site’s sea conditions are examined.

### 5.1. Site Description

**Figure 13:**
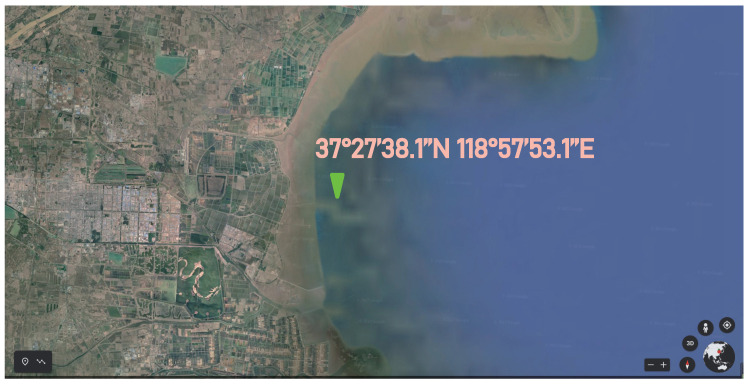
Aerial image (Google Earth) of the experimental site.

The functionality and stability of the sensing system are tested at a wharf in Dongying, Shandong Peninsula, East China (37${}^{{}^{\circ}}$27${}^{\prime }$38.1${}^{\prime \prime }$ N, 118${}^{{}^{\circ}}$57${}^{\prime }$53.1${}^{\prime \prime }$ E). Figure 13 shows an aerial photograph of the trial site given by Google Earth. The experimental platform is roughly 2.8 km off the coast in the Bohai Sea, the average water depth of which is about 18 m. There are no barriers near the testing location, therefore multipath signals from rocks and tiny islands are efficiently avoided. As a result, this location is appropriate for monitoring sea conditions.

**Figure 14:**
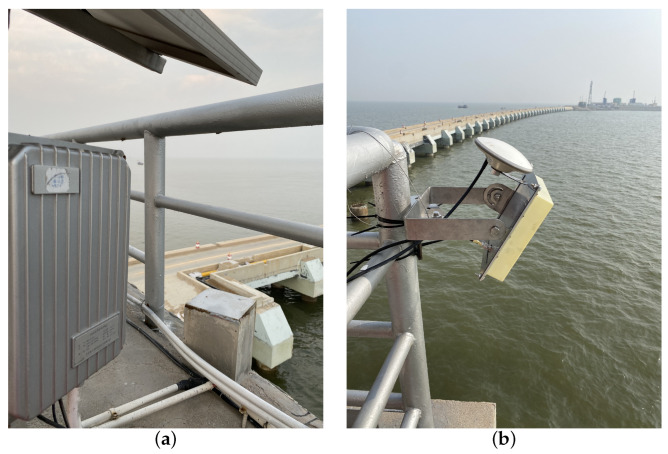
GNSS-R experiment setup at Dongying, China. (**a**) Sensor prototype mounted on the sea site; (**b**) Photograph of the side-looking antennas, including the RHCP antenna and LHCP antenna.

The direct signals are received using an RHCP L1/B1 antenna in the zenith direction. The reflected signals are received using an LHCP L1/B1 antenna slanted with an azimuth angle of 125${}^{{}^{\circ}}$ and an elevation angle of 45${}^{{}^{\circ}}$ towards the sea surface, as shown in Figure 14. The antennae are mounted on the wharf at a height of around 14.5 m above sea level. The wharf is attached with the supplied equipment case was installed on the wharf.

### 5.2. Test Campaign Results

The data obtained from the field is saved in Local Server Group, and the field equipment is gathered every 5 min for 1 min each time. These 1-min data blocks will be processed as part of the data processing.

**Figure 15:**
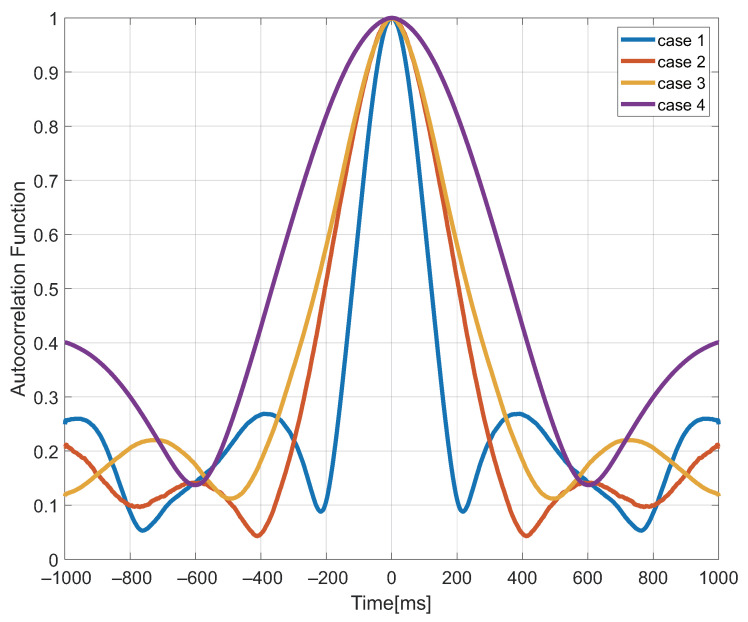
ICF correlation time curve in 4 cases.

From a data transmission standpoint, all data is fully uploaded, the on-site computation time is less than 1 min, and a set of wind and wave values may be communicated every 5 min. The wind speed and significant wave height during 5 min are represented by the default using data acquired within 1 min. That is to say, the system’s resolution is 5 min, which is sufficient for monitoring the sea surface. Furthermore, the system eliminates the need for data post-processing by placing retrieval equipment on-site, allowing for real-time transmission of sea surface wind and wave values to the server for viewing by users. The empirical parameters were determined with a nonlinear least squares approach using the ICF correlation time from each 1-min block of data in the 25-day period, as well as the wind speed and SWH value from corresponding to these periods, to identify the best fit to these values. Figure 15 shows the ICF correlation curve after Gaussian fitting for four instances using PRN 4 GEO satellite of Beidou as an example. The ICF correlation times for the four cases in the figure are 151 ms, 247 ms, 326 ms, and 539 ms, respectively. The final sea surface remote sensing results can be inverted using the model discussed in the previous chapter by fitting the sea surface wind speed and effective wave height corresponding to different ICF correlation times.

Wind speed in situ data is provided by the hydrometeorological automatic station, which is associated with the local weather station. There should be no distance difference because the GNSS-R system and the station’s equipment are installed on the same building. The sea surface wind speed data comes from the propeller wind speed sensor, which outputs data every 1 min with a measurement error of (0.2 + 5% × the measured value) meters. The wind speed sensor is around 10 m above ground level. SWH in situ data is collected on an artificial island less than 10 km from the data collection site. The SWH data comes from the acoustic wave instrument, which outputs data every 30 min with a measurement error of (0.2 + 5% × the measured value) meters.

**Figure 16:**
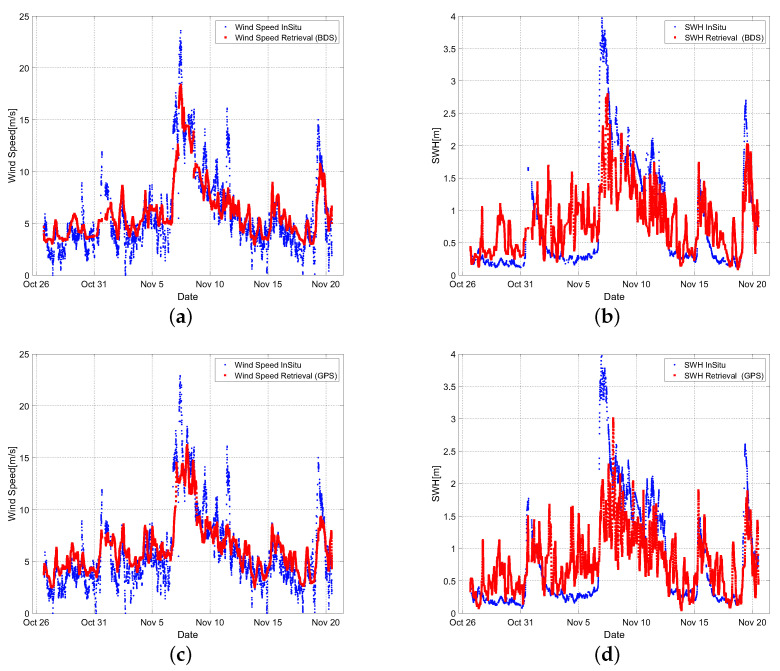
Retrieval results using Beidou system and GPS system, respectively: (**a**) Wind speed results using Beidou system; (**b**) Significant wave height results using Beidou system; (**c**) Wind speed results using GPS system; (**d**) Significant wave height results using GPS system.

First, the Beidou and GPS systems were investigated separately, with the results shown in Figure 16. The RMSE of the Beidou system wind speed/wave height is 2.12 m/s and 51.72 cm, whereas the GPS system has an RMSE of 2.44 m/s and 57.68 cm. The whole data is then filtered, and the data is picked according to the geometry to select satellites with a high signal-to-noise ratio and proper elevation angle for retrieval, with the result serving as the sea surface wind speed and significant wave height retrieval result at this moment.

The trend of sea surface wind speed and significant wave height for 25 days is given after smoothing the comparison of the retrieved results and the in situ measured data in the time series. The RMSE of wind speed is 1.82 m/s, while the RMSE of SWH is 0.38 m, according to calculations, which is better than one single global navigation system with the smoothing processing and data selection. The goal of monitoring sea conditions has been accomplished from the standpoint of total data retrieval. Among them, a strong wind swept across the location on 7 November, and the wind speed swiftly climbed from 1 m/s to more than 20 m/s, showing that the entire system was running correctly and high wind speed/SWH could be detected.

According to Figure 16, wind speed retrieval accuracy is better than SWH accuracy, and when the wind speed is less than 10 m/s, the effect is better. The in situ data has different cycles in our data processing. In order to match the ICF correlation time one by one, the interpolation approach is used for the in situ SWH values. This could be one of the reasons why the retrieved SWH does not appear to be as excellent as the sea surface wind speed. Furthermore, the SWH in situ data gathering station is located far from the GNSS-R system. This could be another cause for the poor performance of SWH retrieval. However, regardless of wind speed or SWH, there is a phenomenon of poor accuracy at very high and very low wind speeds. It can be explained that the predicted parameters of the fitting curves at high wind speeds differ from those at low wind speeds. The fraction of samples with high wind speed is minimal from the perspective of the full data segment, and the fitting effect is poor, which is also why the inversion effect is poor at high wind speed.

The aforementioned results were acquired by processing the reflected signal, as previously stated. The goal of this paper’s study is to develop and test the complete system, particularly the hardware, therefore it only focuses on the inversion using the technique given above to obtain the findings. The prototype was found to be effective in providing reflected signal measurements that aid in real-time monitoring of sea surface conditions during the test.

## 6. Conclusions

The design and development of a real-time monitoring system for sea conditions based on the GNSS-R concept is presented in this work. Because this GNSS passive radar system is intended for long-term field observation, the design of the entire system was bound from the start by structure and mobility.

The sensor has four synchronized RF channels for receiving direct and reflected GNSS signals via RHCP and LHCP polarizations, respectively. The RF section is coupled to an FPGA and DSP stage with a commercial embedded microprocessor for direct and reflected signal processing, respectively. It contains 12 reflection channels of data as an output file from the hardware platform (6 for the Beidou system and the rest for GPS system). The data includes I and Q values for direct and reflected signals, which may be calculated after processing, as well as basic satellite information such PRN number and elevation angle. The data sent to Raspberry Pi is standardized and can be analyzed in real time. Based on the technique described above, the sea surface wind speed and SWH are obtained. Local servers, in addition to the hardware, are intended to accept data from the scene based on the APIs that are used for data exchange. The sensor was thoroughly tested in the lab, allowing for an assessment of its performance in a real-world setting. The sensor’s capacity to receive reflected signals was successfully proven.

Scalability and stability are the strengths of the designed sensor system. These benefits have been confirmed by field testing findings from the deployment of the installed sensor case so far at a coastal location in Shandong, China. The results of the field testing reveal that the implemented sensor has a high level of stability and fits the requirements for long-term sea conditions monitoring. An experiment for wind speed and SWH validation over a longer time period is planned for future research, with the aim of proposing a better approach to improve retrieval results. The research will continue for further data analysis, which will be undertaken, and the findings of the analysis will be utilized to provide users with new applications and monitoring maintenance may ultimately be accomplished.

## Figures and Tables

**Table 1:** Parameters of the LHCP antenna used in this paper.

Parameter	Value
Antenna standing wave ratio	1.4 dB
Antenna gain	12 dB
Antenna front to back ratio	24 dB
Axial ratio	2 dB
Polarization isolation	20 dB
Low noise amplifier LNA gain	34 dB
DC	3–5 V 40 mA

## Data Availability

Not applicable.
